# Calmodulin disruption impacts growth and motility in juvenile liver fluke

**DOI:** 10.1186/s13071-016-1324-9

**Published:** 2016-01-27

**Authors:** Erin M. McCammick, Paul McVeigh, Paul McCusker, David J. Timson, Russell M. Morphew, Peter M. Brophy, Nikki J. Marks, Angela Mousley, Aaron G. Maule

**Affiliations:** Microbes & Pathogen Biology: Institute for Global Food Security, School of Biological Sciences, Queen’s, University Belfast, Medical Biology Centre, 97 Lisburn Road, Belfast, BT9 7BL UK; Institute of Biological, Environmental and Rural Sciences, Aberystwyth University, Penglais, Aberystwyth, Ceredigion, SY23 3FL UK

**Keywords:** *Fasciola hepatica*, Calmodulin, Flukicide target, RNAi, Growth phenotype, Motility phenotype

## Abstract

**Background:**

Deficiencies in effective flukicide options and growing issues with drug resistance make current strategies for liver fluke control unsustainable, thereby promoting the need to identify and validate new control targets in *Fasciola* spp. parasites. Calmodulins (CaMs) are small calcium-sensing proteins with ubiquitous expression in all eukaryotic organisms and generally use fluctuations in intracellular calcium levels to modulate cell signalling events. CaMs are essential for fundamental processes including the phosphorylation of protein kinases, gene transcription, calcium transport and smooth muscle contraction. In the blood fluke *Schistosoma mansoni*, calmodulins have been implicated in egg hatching, miracidial transformation and larval development. Previously, CaMs have been identified amongst liver fluke excretory-secretory products and three CaM-like proteins have been characterised biochemically from adult *Fasciola hepatica*, although their functions remain unknown.

**Methods:**

In this study, we set out to investigate the biological function and control target potential of *F. hepatica* CaMs (*Fh*CaMs) using RNAi methodology alongside novel in vitro bioassays.

**Results:**

Our results reveal that: (i) *Fh*CaMs are widely expressed in parenchymal cells throughout the forebody region of juvenile fluke; (ii) significant transcriptional knockdown of *FhCaM1-3* was inducible by exposure to either long (~200 nt) double stranded (ds) RNAs or 27 nt short interfering (si) RNAs, although siRNAs were less effective than long dsRNAs; (iii) transient long dsRNA exposure-induced RNA interference (RNAi) of *FhCaM*s triggered transcript knockdown that persisted for ≥ 21 days, and led to detectable suppression of *Fh*CaM proteins; (iv) *FhCaM* RNAi significantly reduced the growth of juvenile flukes maintained in vitro; (v) *FhCaM* RNAi juveniles also displayed hyperactivity encompassing significantly increased migration; (vi) both the reduced growth and increased motility phenotypes were recapitulated in juvenile fluke using the CaM inhibitor trifluoperazine hydrochloride, supporting phenotype specificity.

**Conclusions:**

These data indicate that the Ca^2+^-modulating functions of *Fh*CaMs are important for juvenile fluke growth and movement and provide the first functional genomics-based example of a growth-defect resulting from gene silencing in liver fluke. Whilst the phenotypic impacts of *Fh*CaM silencing on fluke behaviour do not strongly support their candidature as new flukicide targets, the growth impacts encourage further consideration, especially in light of the speed of juvenile fluke growth in vivo.

**Electronic supplementary material:**

The online version of this article (doi:10.1186/s13071-016-1324-9) contains supplementary material, which is available to authorized users.

## Background

*Fasciola* spp. liver flukes cause fasciolosis, a disease that impacts heavily on farmed animal health and food security, and is of growing importance as a human neglected tropical disease [1–3]. There are only a handful of available flukicides with which to combat these infections, and their utility is being eroded by the increasing occurrence of drug resistance in both veterinary and human infections [4, 5]. Triclabendazole is the only flukicide with significant activity against juvenile liver fluke [6], the life-cycle stage that causes host tissue damage during its migration from the gut lumen, through the intestinal mucosa and hepatic parenchyma to the bile ducts. Novel flukicides, especially those with activity against juvenile fluke, are a pressing need.

Calcium ions (Ca^2+^) are ubiquitous messengers with an array of roles in physiological and metabolic pathways. External stimuli or signals often lead to variations in intracellular Ca^2+^ levels that regulate processes such as transcription, neurotransmitter release and muscle function [7]. The range of responses that can result from Ca^2+^ signalling are a direct result of the temporal and spatial organisation of the signal [8]. In many cases, particularly longer acting responses, effector proteins cannot bind Ca^2+^ directly and require an intermediate Ca^2+^ sensing protein in order to initiate a biological response.

Calmodulin (CaM) is the most studied Ca^2+^ sensing protein in mammals and can act as a monomer, or as a subunit in multimeric proteins [9]. Within its dumbbell shape it possesses two globular domains, each with the ability to bind two Ca^2+^ ions (four binding sites per CaM), connected by a flexible linker region. Normally, cytosolic Ca^2+^ levels are low, but even a small rise in cytosolic [Ca^2+^] can trigger Ca^2+^ binding and a conformational change in CaM that facilitates the interaction between the Ca^2+^/CaM complex and target proteins [10]. The cooperative binding properties of CaM mean that small increases in cytosolic Ca^2+^ can be translated, via CaM and other calcium sensors, to a multitude of biological processes such as transcription, muscle activity, neurotransmission, cytoskeletal assembly/reorganisation and cell death [10, 11]. The key role played by CaM in diverse biological processes makes it an appealing target for the control of pathogens. Indeed, the disruption of Ca^2+^ regulation is believed to be key to the mode of action of praziquantel, used for the control of *Schistosoma mansoni* and various cestodiases [12].

Despite this interest, CaMs in flatworms are still quite poorly understood. CaM-like proteins have been identified in several important fluke species. Two CaMs have been identified and characterised from *S. mansoni* [13], showing high sequence identity (>98 %) to CaMs from mammals. Silencing these schistosome CaMs using RNA interference (RNAi) resulted in shorter worms in larval stages and somatic muscle contraction/dilation in adults, while CaM antagonists inhibited miracidial transformation in a concentration dependent manner [13, 14]. Previously, a CaM inhibitor was shown to inhibit egg hatching and miracidial transformation in *S. mansoni* [15, 16]. A *Clonorchis sinensis* CaM was shown to bind Ca^2+^ and to be expressed in diverse developmental stages including the egg, metacercaria and adult where it was localised in the intestine, pharynx and tegument [17]. A divergent CaM-like protein has been reported in *Schistosoma japonicum* [18] and three CaM-like proteins have been identified and biochemically described in *Fasciola hepatica* [19, 20]. The first, *Fh*CaM1, displays high identity to mammalian CaM with only two amino acids differing. *Fh*CaM2 and *Fh*CaM3 are more divergent, displaying lower sequence similarity, as well as distinct biochemical and ion binding properties. This prompted the hypothesis that they are involved in different pathways or perform roles distinct from that of *Fh*CaM1 in the organism. All three fluke CaMs were predicted to adopt a calmodulin-like fold and shown to bind Ca^2+^. Immunocytochemistry failed to detect *Fh*CaM1 but localised *Fh*CaM2 and *Fh*CaM3 to eggs and vitelline cells in adult worms [19, 20].

This study builds upon our description of RNAi dynamics in juvenile fluke [21], employing RNAi methods to investigate the biological functions of *Fh*CaM1, *Fh*CaM2 and *Fh*CaM3 in juvenile *F. hepatica*. We show that *Fh*CaM2 and *Fh*CaM3 are expressed widely in parenchymal cells of juvenile fluke and that *FhCaM* RNAi inhibits growth and modulates motility, phenotypes that can be recapitulated using a CaM antagonist drug. These data provide the first functional data for liver fluke calmodulins, including the first description of an RNAi-induced growth phenotype in liver fluke, suggesting that drugs capable of targeting CaM’s role in growth/development might represent appealing new additions to our flukicidal arsenal.

## Methods

### Parasites and maintenance

*F. hepatica* metacercariae (Oregon strain) were purchased from Baldwin Aquatics Inc. (Monmouth, Or, USA) and stored in distilled water at 4 °C. These metacercariae were supplied without their outer cyst walls, with excystment performed as described previously [22]. Worms were transferred, as they excysted, into RPMI 1640 supplemented with antibiotic/antimycotic (100 U/ml penicillin, 100 μg/ml streptomycin and 25 μg/ml amphotericin B, Life Technologies, Carlsbad, USA), before further processing as described below. At all stages of these experiments, excysted juvenile fluke were handled under aseptic conditions and maintained at 37 °C in a humidified 5 % CO_2_ atmosphere.

### Genomic organisation of CaM encoding genes

The *F. hepatica* genome [23] was queried using tBLASTn analysis for the translated sequences of *FhCaM1*, *FhCaM2*, and *FhCaM3*. Returned hits were assessed for bit score and E-value and manually assembled from the parent scaffold using Artemis to decipher exon organisation [24]. Contigs were aligned pairwise with the corresponding query sequence to ensure identity.

### Immunocytochemistry

Freshly excysted fluke were fixed for 4 h at room temperature in 4 % paraformaldehyde in 0.1 M Phosphate Buffered Saline (PBS prepared from tablets; Sigma Aldrich, St Louis, USA). Primary antisera were raised in rabbits against each of the following: recombinant human-CaM (structurally similar to *Fh*CaM1 and differs by two amino acids; antiserum shown previously to cross-react with *Fh*CaM1; Sigma-Aldrich); *Fh*CaM2; and, *Fh*CaM3 [19, 20]. Incubations in primary antisera were performed at 1/200 dilution (diluted in antibody diluent, AbD; 0.1 M PBS, 0.1 % (v/v) TritonX-100, 0.1 % (w/v) bovine serum albumin, 0.1 % (w/v) NaN_3_) for two days. Samples were then incubated consecutively in secondary antibody (fluorescein isothiocyanate (FITC)-labelled goat anti-rabbit; 1:100) overnight, and then overnight in tetramethylrhodamine isothiocyanate (TRITC)-labelled phalloidin (200 ng/ml), both diluted in AbD. Incubations were performed at 4 °C unless stated otherwise, and at least three washes in 1 M NaCl in PBS, 0.1 % TritonX-100, 0.2 % Tween-20, were performed between incubations. Samples were mounted in Vectashield (Vector Laboratories, Peterborough, UK) and viewed on a Leica TCS SP5 confocal scanning laser microscope.

### CaM RNAi methodology

*CaM* transcript dsRNA templates were amplified from PCR amplicons representing the coding sequence of each *CaM* gene. These coding sequences were amplified using the following primers, designed against the GenBank accession numbers provided: *FhCaM1* [AM412546], forward 5’-atggctgatcaactcacagaaga-3’, reverse 5’-tcattttgcagtcatcattttcac-3’; *FhCaM2* [AM412547], forward 5’-ggtgcaccatctctcacaag-3’, reverse 5’-ttgcgcagaagagtcaagaa-3’; *FhCaM3* [JQ792169], forward 5’-atggctgactttgatatggatca-3’, reverse 5’-ctacaggtattcgtatgcaaattcc-3’. Templates for dsRNA constructs were amplified from these primary amplicons in a nested PCR reaction, using primers labelled (on 5’ end) with a T7 RNA polymerase promoter sequence (5’-taatacgactcactatagggt-3’), to create templates labelled with T7 in either sense or antisense directions (*FhCaM1*, forward 5’- cagaagagcagatcgcagaa-3’, reverse 5’-aattcggggaaatcaatggt-3’; *FhCaM2*, forward 5’- agatgccacggaactgaaag-3’, reverse 5’-aaatgtacccgtcgccatta-3’; *FhCaM3*, forward 5’- atgacgctgtacgacaccaa-3’, reverse 5’-agtggtgggcacaacgatac-3’). All PCR amplicons were sequence confirmed [25]. This approach generated dsRNA templates for *FhCaM1*, *2,* and *3* of 190 bp, 221 bp and 210 bp, respectively. Exogenously derived (i.e. non-worm) negative control dsRNA templates (bacterial neomycin phosphotransferase; [U55762]) were generated from the cloning vector pEGFP-NI (Clontech Laboratories Inc., Saint-Germain-en-Laye, France), using the following primers: forward, 5’-ggtggagaggctattcggct-3’; reverse: 5’-ccttcccggttcagtgacaa-3’. These templates were used to generate double stranded (ds)RNAs as described in McVeigh et al. [21].

Short interfering (si)RNAs (27mer) for each *Fh*CaM were designed and synthesised by [26]; a randomised siRNA control was also used. Target sequences for these siRNAs were as follows: *FhCaM1,* 5’-augacugucccaagcuccuucguggua-3’; *FhCaM2,* 5’-uauguaagacugaucucaauucuuccg-3’; *FhCaM3*, 5'-ucccugucacuaacccuccucuuuguu-3’; and negative control siRNA sequence *siCTRL*, 5’-cttcctctctttctctcccttgtga-3’.

In order to expose juvenile fluke to RNAi trigger molecules, fluke were soaked (20 NEJs per replicate for transcriptional analysis, 10 NEJs per replicate for phenotype assays and 50 NEJs per replicate for western blot analysis) in 50 μl of 100 ng/μl (dsRNA), or 50 ng/μl (siRNA) solution of *FhCaM* ds/siRNA dissolved in RPMI 1640, alongside both untreated (no ds/siRNA) and negative control (dsCTRL/siCTRL) treatments, with all soaks performed at least in triplicate. Soaks were performed for 4 h at 37 °C, in 5 % CO_2_. After this 4 h incubation, 250 μl maintenance media was added (RPMI 1640, containing antibiotic/antimycotic and supplemented with 20 % foetal bovine serum (FBS)). Worms were maintained in this medium for up to 21 days with a change of media once every 2–3 days. During media changes worms were observed using light microscopy for obvious phenotypic changes (aberrant morphology or motility), or imaged for measurements as described below.

### Transcript quantification

Transcript abundance was assessed in NEJs maintained in the presence (+) and absence (−) of serum in the maintenance media at 0-, 1-, 3-, 5- and 7-days. Note that 97 % of juvenile fluke are viable by day 7 in RPMI and 100 % are viable in RPMI supplemented with serum. Parasites remain viable and motile for several weeks under these conditions (data not shown). For RNAi experiments, transcript abundance was assessed following maintenance at 3-, 14- and 21-days post dsRNA (or siRNA) exposure, by quantitative PCR (qPCR). Poly-adenylated mRNA, extracted from treatment groups (20 NEJs per replicate) using Dynabeads mRNA direct Kit (Life Technologies, Carlsbad, USA), was used to synthesise cDNA (High Capacity RNA-to-cDNA kit, Life Technologies) following DNase treatment (Turbo DNA-free, Life Technologies). qPCRs were performed on a Rotor-Gene Q 5-plex HRM PCR system (Qiagen), using the following gene-specific primers: *FhCaM1*, forward 5’- tggtaacgggaccattgatt-3’, reverse 5’- ttatcgaacacacggaatgc-3’; *FhCaM2*, forward 5’- atcgcggcaatcttgataat-3’, reverse 5’-ttgcgcagaagagtcaagaa-3’; *FhCaM3*, forward 5’- agcttcggaactgcacaaat-3’, reverse 5’-cgttgtcaccatctttgtcg-3’. Target primers were amplified alongside the glyceraldehyde 3-phosphate dehydrogenase (GAPDH) reference transcript (*FhGAPDH* [GenBank; AY005475]), forward 5’-ggctgtgggcaaagtcattc-3’, reverse 5’- agatccacgacggaaacatca-3’). Primers were added to 8 μl PCR reactions at a final concentration of 1.25 μM, including cDNA diluted 1:1 with water, and FastStart SYBR Green Master (Roche Applied Science). Cycling parameters employed were a 10 min “hot-start” at 95 °C, followed by 40 cycles of 95 °C 10 s, 60 °C 15 s, 72 °C 30 s. All PCRs were performed in triplicate, and included no-template controls and melt-curve analyses as standard. Relative expression analysis employed Pfaffl’s Augmented ΔΔCt method [27] (which normalises expression in each sample relative to the untreated control, standardised to a GAPDH reference amplicon), with amplification efficiencies of individual reactions calculated using Real Time PCR Miner software [28, 29]. This analysis method produces a ratio of target transcript abundance relative to the untreated control, which was then converted into percentage change (i.e. where a value of 100 % represents no change). These data (mean % transcript abundance relative to *FhGAPDH* reference ± SEM) are plotted in bar graphs in the qPCR figures. All datasets were normally distributed according to the Kolmogorov-Smirnov test and were, therefore, analysed using ANOVA with Tukey's or Dunnett’s multiple comparison post hoc test. Statistical significance was determined relative to the effects of negative control treatments (dsCTRL or siCTRL) on target gene expression.

### Analysis of CaM protein

Protein abundance was assessed after RNAi treatments and maintenance in the presence of serum at days −7 and −14 post dsRNA, using western blot methods. Protein was extracted from tissue matched (50 NEJs) homogenised treatment groups in 50 μl radioimmunoprecipitation assay (RIPA) buffer (Sigma-Aldrich), with blots performed as described previously [30]. Following protein transfer onto nitrocellulose membrane, the top half of each membrane (relatively high molecular weight proteins) was probed with rabbit anti-actin (1:400) (Sigma Aldrich) as a loading control. The bottom half (relatively low molecular weight proteins) was probed with rabbit anti-*Hs*CaM1 (1:1000), rabbit anti-*Fh*CaM2 (1:1000) or rabbit anti-*Fh*CaM3 (1:1000); these incubations were performed in parallel. Secondary antibody was an alkaline phosphatase conjugated goat-anti-rabbit (Sigma Aldrich), used at 1:1000 dilution. Development was performed in nitro blue tetrazolium chloride/5-bromo-4-chloro-3-indolyl-phosphate, toluidine-salt (NBT/BCIP) solution.

### CaM antagonists

We used the CaM antagonists, trifluoperazine hydrochloride (TFP) and N-(6-aminohexyl)-5-chloro-1-naphthalenesulfonamide hydrochloride (W7) (both Sigma Aldrich) [31]. Drugs were dissolved in 100 % dimethylsulfoxide (DMSO) and added to maintenance media such that the final DMSO concentration was 0.1 %. Two methods of administration were employed: (i) short term exposure performed in RPMI 1640 for 18 h at final concentrations 10, 5, 2 and 0.5 μM, including vector (DMSO) only control and untreated control; and (ii) long term exposure performed in RPMI + 20 % FBS over 7 days in constant presence of TFP with final concentrations at 5, 1, 0.5, 0.1, 0.05 μM plus controls as outlined above.

### Phenotypic analyses

Following RNAi and drug treatments, we assayed for changes in both motility/migration and growth phenotypes. All analyses were performed in an operator-blinded fashion, on coded samples. Analysis of worm motility was performed on drug-treated (18 h) worms by capturing videos (30 s), and manually counting the numbers of worms displaying full, “normal” motility (i.e. waves of circular muscle contraction travelling from anterior to posterior) during the analysis period. Analysis of migration was performed on both drug treated and RNAi-treated worms. Migratory capacity under these experimental conditions was assessed using an agar dispersal assay, in which worms were placed into an agar substrate (0.2 % cell culture grade agar (Sigma Aldrich) in HEPES-buffered Ringer’s (123 mM NaCl, 5 mM KCl, 1.6 mM CaCl_2_, 20 mM HEPES, 11.1 mM d-glucose; pH 7.4)) at the centre of a petri dish. The proportion of worms migrating beyond an empirically determined radius (5 mm) was then recorded over a 3 h time-course using a measuring grid under a dissecting microscope.

Growth analyses were performed on both drug and RNAi treated worms (and controls). Images captured using a Leica MZ 12.5 stereomicroscope and Unibrain Fire-i digital camera were subject to measurements of length and area using the measurement tools in ImageJ software [32]. Growth and motility assay data were analysed using one-way ANOVA with Dunnett’s post hoc test, analysing significance of treatments relative to the negative control ds/siRNA treated worms (RNAi experiments), or vehicle control treated worms (drug experiments).

## Results and Discussion

This study reports functional characterisation of three CaM-like proteins in *F. hepatica* juveniles. Although the in vitro biochemistry of the three *Fh*CaM recombinants, and their adult localisations have been reported previously [19, 20], functional genomics analyses have not been performed on CaMs in any *F. hepatica* life-cycle stage. Motivated by reports of CaM RNAi phenotypes in other helminths [13, 33] we focused here on assessing CaM function in juvenile fluke, given the amenability of this life-cycle stage to our target validation platform for fluke that encompasses RNAi methods [21, 22, 34] and diverse functional assay platforms. A primary motivation for focusing on CaM, a protein intimately involved in cellular Ca^2+^ signalling processes, is the fact that Ca^2+^ signalling and regulation play key roles in neuromuscular function, which represents a well-established repository of targets for empirically-discovered anthelmintics [35]. Indeed, the disruption of Ca^2+^ regulation by praziquantel, the drug of choice for the treatment of schistosome and tapeworm infections, directly highlights the validity of interventions disrupting Ca^2+^ signalling/regulation. Whilst previous work on liver fluke CaMs focussed on in vitro function and adult stage expression, our focus was CaM expression and biological importance as a measure of control target validity in juvenile fluke. It is well established that effective flukicides need to target both the migrating juvenile and adult fluke stages. In this way our data inform CaM expression, function, and potential as a therapeutic target for liver fluke control.

### Genomic organisation

Analysis of hits collected from querying the genomic dataset with pre-confirmed *Fh*CaM1, *Fh*CaM2 and *Fh*CaM3 protein sequences revealed that the *FhCaM1* gene exists in at least two intronless copies on scaffold 932: one full length with an S18F substitution, and one missing the initial 18 amino acids due to an undefined region on the scaffold (Additional file 1: Figure S1). A third copy of *FhCaM1* is present on scaffold 210 (exists as 4 exons) with a stop codon (TAG) in place of the initiating methionine. This could represent a sequencing error or the premature stop codon may indicate that the third *FhCaM1* copy is a pseudogene that would not be translated but could play alternative regulatory roles. *FhCaM2* is present as a single copy on scaffold 2277. Four exons have been identified with possible variants for the amino acid at position 60 (N60S or N60D). The initiating methionine is also absent from the genome sequence of *FhCaM2*, likely due to an artefact of genome assembly as this sequence has been characterised and the initiating methionine confirmed to be present experimentally [19]. *FhCaM3* is also represented by a single copy on scaffold 1560 where the full gene presents as three exons with an apparent duplication of exon 3 (amino acids 71–131) within the scaffold. This duplication likely represents a sequence assembly artefact, as the surrounding non-CaM coding sequence is identical.

These data show that distinct genes code for the CaM proteins. Due to the current annotation status of the genome, we have no data on the relative chromosomal positions of these genes. *Schistosoma mansoni* has also been shown to possess two distinct CaM genes, although they exhibit high (>99 %) sequence identity relative to each other [13].

### FhCaMs are abundantly expressed in parenchymal cells of juvenile liver fluke

**Fig. 1:**
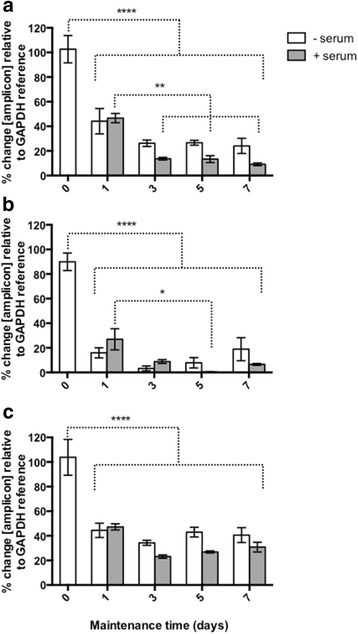
Expression of *FhCaM1* (**a**), *FhCaM2* (**b**) and *FhCaM3* (**c**) during maintenance of juvenile *Fasciola hepatica* +/− serum for 7 days in vitro. CaM transcript abundance was assessed by qPCR at 1, 3, 5, and 7 days and normalised against the abundance of respective transcripts in 0 day juvenile fluke. Data represent mean ± SEM of percentage changes in target transcript abundance relative to a *GAPDH* reference transcript. Each bar represents data from at least five treatment replicates, 20 worms per replicate. Statistical analyses were performed using One Way ANOVA with Tukey’s post hoc test. *, *P* < 0.05; **, *P* < 0.01; ***, *P* < 0.001; ****, *P* < 0.0001

Juvenile liver fluke were maintained in vitro under two conditions: (i) RPMI media where the worms survive in a steady state with no growth or development, and (ii) RPMI supplemented with bovine serum which allows the worms to grow in size, with visible development of the gut [36]. CaM transcript abundance was assessed in worms maintained for 1-, 3-, 5- and 7-days and compared to respective transcript levels at day 0. *FhCaM1*, *FhCaM2* and *FhCaM3* were expressed in the juvenile worms and displayed a significant decrease in transcript abundance (>50 % reduction) compared to those observed at day 0, irrespective of maintenance conditions within the time assayed (Fig. 1). This may occur because upon excysting from a quiescent intermediate stage, juvenile worms may produce significant amounts of gene transcripts in preparation for the migratory stage of the life-cycle. The trends from day 1 onwards show a further significant diminution of *FhCaM1* and *FhCaM2*, with *FhCaM3* remaining relatively stable. Down regulation of *FhCaM1* and *2* reflects a common transcriptional pattern, where a large proportion of genes are downregulated relative to that occurring in metacercariae, through NEJ development towards adulthood [23]. Each of the three CaMs possess distinct ion binding properties and they have been suggested to play different roles. Western blot analyses did reveal that *FhCaM2* and *FhCaM3* are constitutively expressed until day 21 (data not shown) suggesting they are both functional within the juvenile stage of the worms.

**Fig. 2:**
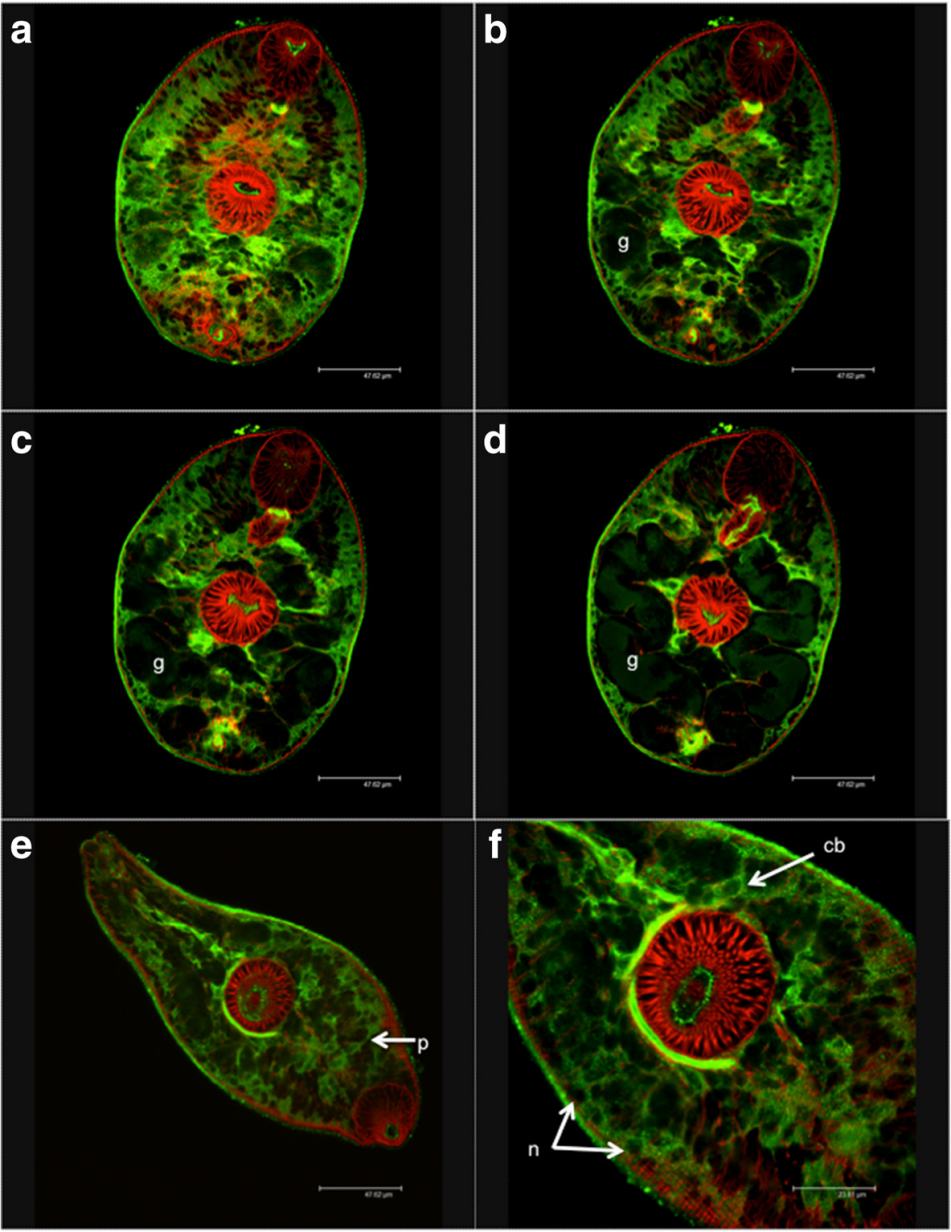
Immunocytochemical localisation of calmodulin 3 (*Fh*CaM3) in juvenile *Fasciola hepatica.* Green immunoreactivity (IR) represents *Fh*CaM3 labelled with fluorescein isothiocyanate, red represents filamentous actin labelled with phalloidin-tetramethylrhodamine isothiocyanate. Images **a**–**d** represent consecutive optical sections taken in the Z-axis (at 1.5 μm intervals), demonstrating the presence of abundant *Fh*CaM3 immunoreactivity (IR) throughout the sub-tegumental, sub-muscular parenchyma. The bifurcated gut (g) can be seen in **b**–**d. e**–**f** show higher magnification images in which *Fh*CaM3 IR is visible within cell bodies (cb) and cellular processes (p), while nuclei (n) remain un-stained. Identical staining patterns were observed for *Fh*CaM2 (not shown). These patterns were visualised in 30 samples

Employing polyclonal antisera previously used in adult worms to localise *Fh*CaM2 to tegumental spines and *Fh*CaM3 to vitelline cells and eggs [20], we demonstrated that *Fh*CaM2 and *Fh*CaM3 are expressed much more widely than previously reported, occurring ubiquitously throughout the parenchymal tissue of juvenile fluke (Fig. 2). Using optical sectioning of stained whole-mount juveniles via confocal microscopy, *Fh*CaM2 and *Fh*CaM3 immunoreactivities (IRs) were detected throughout the parenchyma (i.e. sub-tegumental, non-gut associated tissue) of *F. hepatica* juveniles (*n* = 30 individual worms observed, Fig. 2). *Fh*CaM1-IR was not detected in any of our samples (*n* = 30). Note that the CaM1 antiserum described in the methods did not give any positive IR in juvenile fluke despite multiple experiments, consistent with the results obtained from adult flukes [20]. This would indicate that the *Hs*CaM antiserum could not bind/detect native *Fh*CaM1 despite high levels of sequence conservation (98.6 %). Although Fig. 2 presents only *Fh*CaM3 IR, both *Fh*CaM2 and *Fh*CaM3 antisera displayed identical and similarly diffuse staining patterns. Aided by the counter-staining of filamentous actin using labelled phalloidin, the immunopositive cells were found to be localised below the contractile muscle layers of the body wall (Fig. 2a–d) representing localisation to both cell bodies and cellular processes within the parenchyma which lies below the outer muscular layers and is packed between cells of the gut and other internal organ systems [37]. IR was evident within the cytoplasm of distinct cells, with nuclei remaining unstained (Fig. 2f). Distinct cytoplasmic processes from stained cells were seen extending towards muscle fibres/the muscle layer (Fig. 2c). Cells within the parenchyma include tegumental cell bodies [38] and myocytons (cell bodies) of muscle cells. Myocytons are the non-contractile portion of muscle cells that are located distally to the contractile myofibril [39]. The widespread nature of the localisation shown in Fig. 2 likely reflects the constitutive roles played by CaMs in fluke biology. The essentially identical localisation patterns of *Fh*CaM2 and *Fh*CaM3 make it impossible to suggest functional differences based solely on the microscopy data shown here, although it should be noted that *Fh*CaM2 and *Fh*CaM3 antisera have been tested for specificity in both western blot and ELISA experiments, and show no measurable cross-reactivity [20]. Controls omitting primary anti-CaM sera did not display parenchymal IR, but did exhibit non-specific staining of the outer surface/tegument (data not shown). Therefore, we consider the surface staining visible in Fig. 2 to be non-specific.

### FhCaM RNAi triggers specific suppression of target transcript and protein

**Fig. 3:**
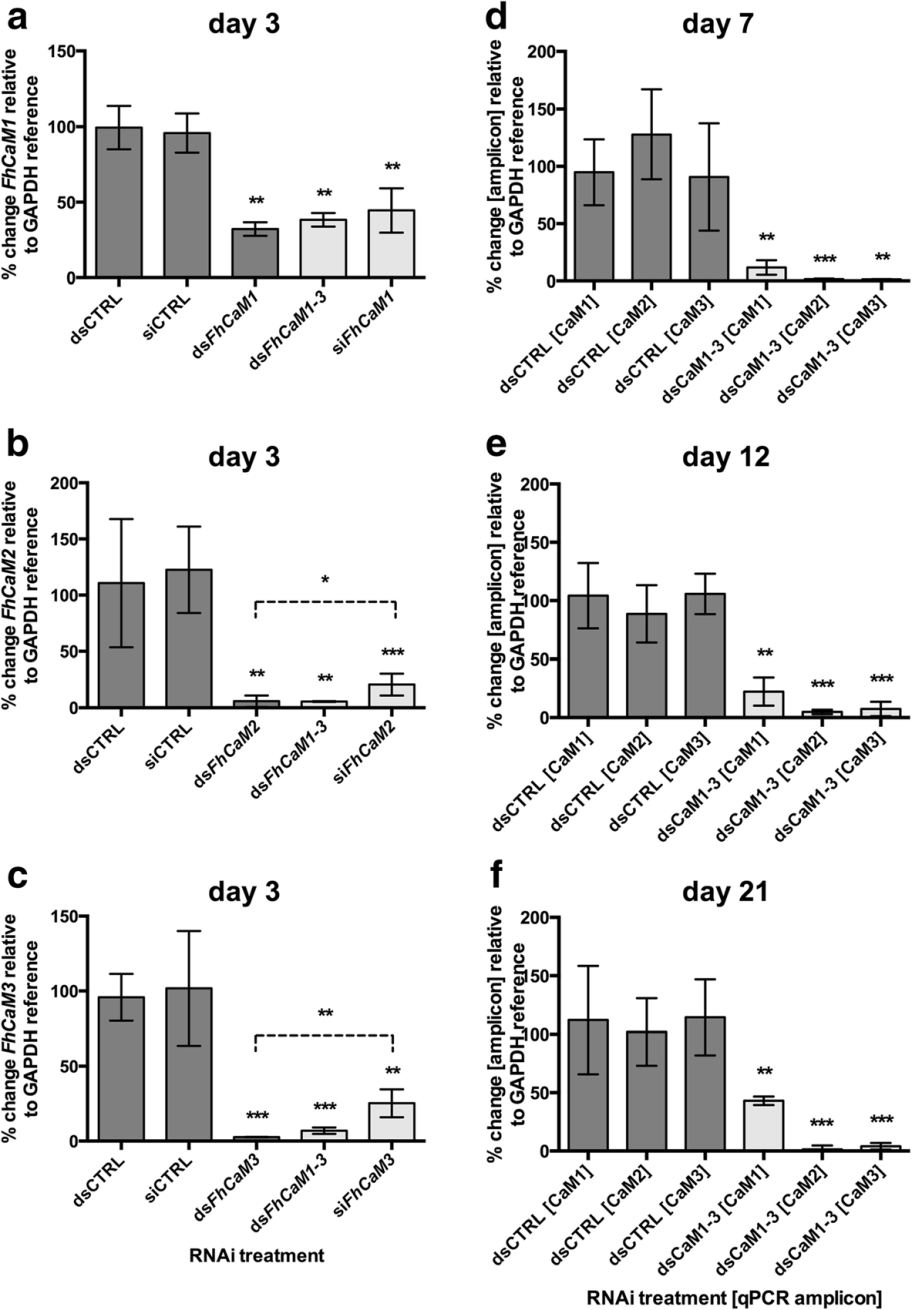
RNA interference (RNAi) of calmodulin (*FhCaM*) transcripts in juvenile *Fasciola hepatica*, as measured by relative quantitative PCR (qPCR). Juvenile fluke exposed to 50 ng/μl short interfering (si)RNA (27 nt) or 100 ng/μl long (~200 nt) double stranded (ds)RNA for 4 h were maintained for a further 68 h (**a**–**c**) or 7 (**d**), 14 (**e**) or 21 days (**f**) before analysis of transcript abundance by qPCR. Over a 72 h time course, impacts of individual CaM dsRNA, a cocktail of *Fh*CaM1-3 dsRNAs, and siRNAs were tested for impact on abundance of *FhCaM1* (**a**), *FhCaM2* (**b**) and *FhCaM3* (**c**) transcripts, alongside negative control treatments (dsCTRL and siCTRL). Effective combinatorial dsRNA treatments were then tested over longer timeframes (**d**–**f**). Data represent mean ± SEM of percentage changes in target transcript abundance relative to a *GAPDH* reference transcript, normalised against the abundance of those transcripts in an untreated control group [27]. Each bar represents data from at least three RNAi treatment replicates, 20 worms per replicate. Statistical analyses were performed using One Way ANOVA with Dunnett’s post hoc test, or Student’s *t*-test (siRNA *vs* dsRNA comparisons in **b**, **c**). *, *P* < 0.05; **, *P* < 0.01; ***, *P* < 0.001

To assess CaM functions in juvenile liver fluke we employed RNAi methods developed in our laboratory [21, 22], based on soaking NEJs in ds/siRNA, followed by maintenance in serum-supplemented RPMI media. We found that exposure to both dsRNA and siRNAs triggered appreciable silencing of *Fh*CaM transcripts, as measured by qPCR. Note that the following data are expressed as the percentage of transcript remaining following RNAi treatment, i.e. where 100 % = no change. Statistical significances are indicated relative to the time/concentration matched negative control treatment. In order to silence the three fluke CaM genes, we began by testing 27 nt siRNAs and long (~200 nt) dsRNAs against each individual target, as well as the three CaM long dsRNAs combined into a cocktail. To assess the relative efficacies of these treatments, each was tested for impacts on CaM transcript knockdown over a 72 h timecourse (i.e. 4 h dsRNA exposure, followed by 68 h maintenance). These initial experiments, showed that target transcript knockdown (in all cases relative to untreated controls) could be triggered by exposure to individual CaM dsRNAs giving significantly reduced levels of expression (ds*FhCaM1 vs FhCaM1* = 32.3 ± 2.6 %, *n* = 3, *P* < 0.01; ds*FhCaM2 vs FhCaM2* = 5.9 ± 3.0 %, *n* = 3, *P* < 0.01; ds*FhCaM3 vs FhCaM3* = 2.5 ± 0.07 %, *n* = 3, *P* < 0.001; Fig. 3a–c) as well as by exposure to a cocktail of all three CaM dsRNAs in combination (dsRNA cocktail = 100 ng/μl final concentration, i.e. each individual dsRNA at 33.3 ng/μl) (ds*FhCaM1-3 vs FhCaM1* = 38.3 ± 2.6 %, *n* = 6, *P* < 0.01; ds*FhCaM1-3 vs FhCaM2* = 5.6 ± 0.2 %, *n* = 3, *P* < 0.01; ds*FhCaM1-3 vs FhCaM3* = 6.9 ± 1.2 %, *n = 3*, *P* < 0.001; Fig. 3a–c). Fig. 3 shows that, in all cases, combinatorial long dsRNA treatments were not significantly different to those of individual dsRNAs i.e. are equally effective. We also tested 27 nt siRNAs against each of the three *Fh*CaMs and found they each induced silencing with significantly reduced levels of expression (si*FhCaM1 vs FhCaM1* = 44.5 ± 6.0 %, *n* = 6, *P* < 0.01; si*FhCaM2 vs FhCaM2* = 20.7 ± 4.0 %, *n* = 6, *P* < 0.001; si*FhCaM3 vs FhCaM3* = 25.3 ± 4.2 %, *n =* 5, *P* < 0.01; Fig. 3a–c). For all three targets the siRNAs appeared less effective than the long dsRNAs; in the cases of *FhCaM2* and *FhCaM3* these differences were statistically significant (Student’s *t*-test: ds*FhCaM2 vs* si*FhCaM2*, *P* < 0.05; ds*FhCaM3 vs* si*FhCaM3*, *P* < 0.01; Fig. 3). Based on these data, we employed dsRNA cocktails representing *FhCaM1-3* in the rest of the experiments reported here. Elsewhere, silencing induced by long dsRNA and siRNA triggers have also been reported to produce robust knockdown for up to 7 days with recovery of transcript levels only occurring in siRNA treatment groups by day 14 [40].

We next analysed the longevity of *CaM* transcript knockdown in vitro over a 3-week period in order to begin to assess the time frame over which functional assays could be run. *CaM* knockdown persisted at significant levels during maintenance (measurements taken at 7, 14 and 21 days post dsRNA exposure; Fig. 3d–f), with transcript levels at day 21 remaining significantly lower than controls (*FhCaM1* = 43.1 ± 4.1 %, *n* = 4, *P* < 0.01; *FhCaM2* = 1.5 ± 1.5 %, *n* = 5, *P* < 0.001; *FhCaM3* = 4.0 ± 1.3 %, *n* = 5, P < 0.001; Fig. 3f). These data showed that CaM transcript knockdown persists throughout this maintenance period; this persistence occurs even in the absence of dsRNA during maintenance, since worms were only exposed to dsRNA during the initial 4 h exposure soak. Such long-term maintenance of knockdown is consistent with our previous observations on RNAi of liver fluke cathepsin B, L and glutathione transferase genes, in which we reported the persistence of knockdown for 21 days following a similar RNAi protocol [21]. Similarly, electro-soaking methods have also shown the persistence of silencing effects up to 21 days [40] and reports from schistosomes illustrate that electroporation-induced RNAi can be maintained for up to 40 days [41]. Such persistent silencing in trematodes, even in the absence of prolonged dsRNA supplementation, indicates the presence of an efficient dsRNA uptake and amplification mechanism and bodes well for the success of putative in vivo RNAi experiments.

**Fig. 4:**
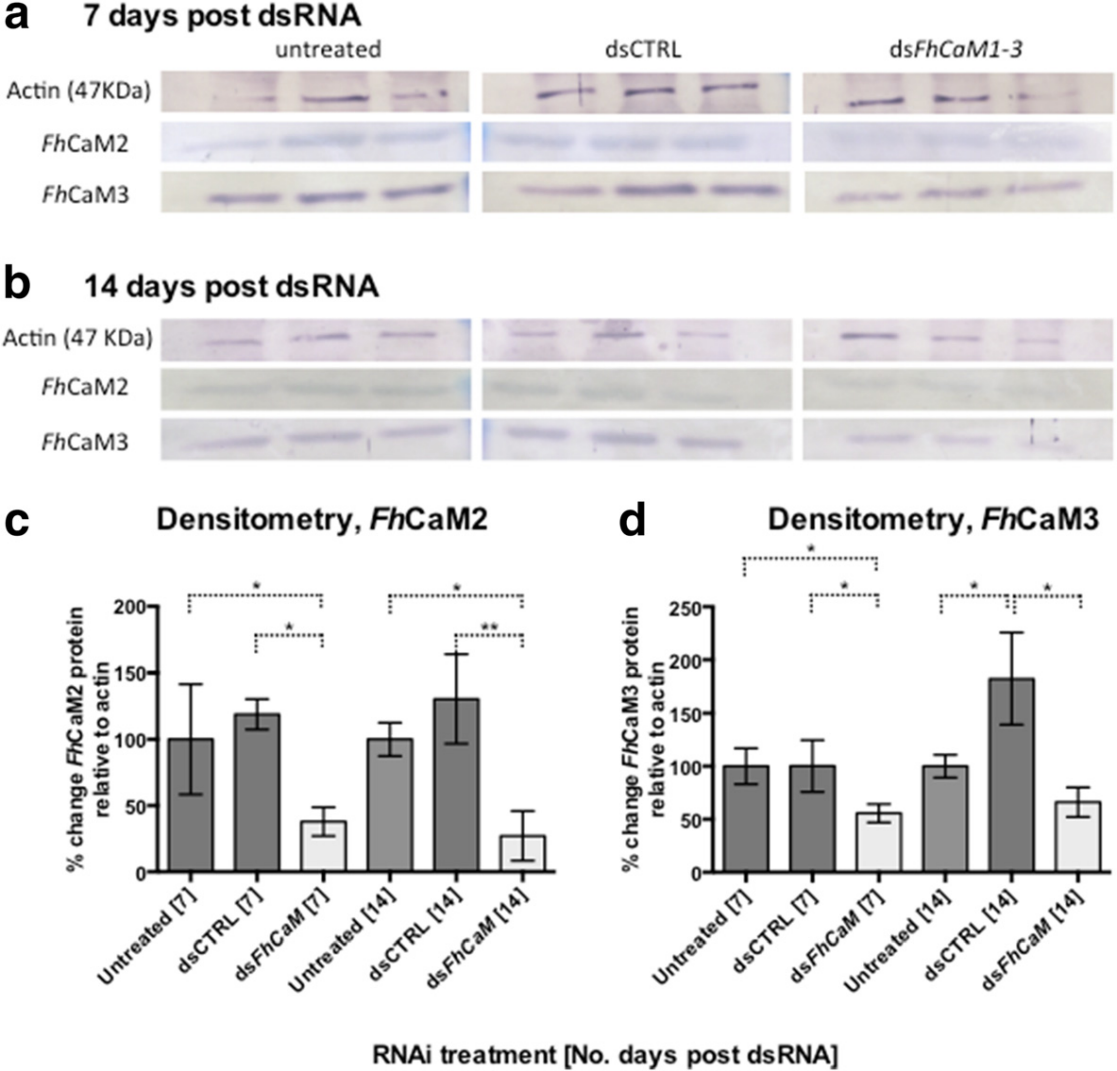
RNA interference (RNAi)-induced suppression of *Fasciola hepatica* calmodulins *Fh*CaM2 and *Fh*CaM3 in juvenile fluke. Western blots were used to measure suppression of both *Fh*CaM2 and 3 relative to a loading control (anti-actin) at both 7 (**a**) and 14 (**b**) days following exposure to CaM double stranded (ds)RNA. All three *Fh*CaM targets were measured in untreated controls, negative control dsRNA treatments (dsCTRL) and a dsRNA cocktail targeting all three fluke CaMs (ds*Fh*CaM1-3). Reduced band density, indicating suppression of target protein, is apparent in *Fh*CaM2- and *Fh*CaM3-probed ds*Fh*CaM1-3 treatments at both 7 and 14 days post dsRNA, as evidenced by densitometric analyses of *Fh*CaM2 (**c**) and *Fh*CaM3 (**d**). Each lane represents a single replicate sample, consisting of protein extracted from 50 NEJs per replicate. *, *P* < 0.05; **, *P* < 0.01

Persistent transcript knockdown over a 3-week measurement period suggested that CaM protein suppression would be detectable during this period, following run-down of target proteins in line with their cellular half-lives. As a guide to the time-point at which functional assays could most effectively be targeted, we performed western blot-based detection of *Fh*CaM2 and *Fh*CaM3 at 7 and 14 days following dsRNA exposure (Fig. 4). Using *Fh*CaM2 and *Fh*CaM3 antisera at days 7 and 14 post dsRNA exposure, these proteins exhibit RNAi-induced suppression relative to time-matched dsCTRL and untreated controls from day 7 onwards (*Fh*CaM2: day 7, 37.9 ± 6.3 %, *n* = 3, *P* < 0.05; day 14, 27.1 ± 13.3 %, *n* = 3, *P* < 0.01; *Fh*CaM3: day 7, 55.6 ± 5.0, *n* = 3, *P* < 0.05; day 14, 66.2 ± 9.9, *n* = 3, *P* < 0.01; Fig. 4). The exception here was *Fh*CaM3 at day 14, which did not show a significant effect compared to untreated control, indicating either recovery from suppression (although *FhCaM3* transcripts remained suppressed beyond this time point), or a technical issue with our measurement method leading to a false negative reading in this case. These data demonstrate that both *Fh*CaM2 and 3 levels were significantly lower in ds*FhCaM1-3* treatments than in either control group at day 7; this difference persists until at least day 14. We therefore proceeded with functional analyses of the biological consequences of CaM RNAi, at time points ≥ 7 days post dsRNA exposure.

### FhCaM RNAi induces motility and growth phenotypes in vitro

Assay selection was guided by our hypotheses that CaMs are involved in worm growth and motility. Previous work has linked CaM function with growth and development of helminths, including reduced growth associated with CaM silencing in both *S. mansoni* sporocysts [13], and in the free-living nematode *C. elegans* [33]. Recently, the silencing of adult *S. mansoni* CaM has implicated the protein in muscular function and resulted in a characteristic somatic contraction/dilation phenotype [14]. Given the persistence of *CaM* transcript knockdown (Fig. 3) and significant protein suppression from day 7 onwards (Fig. 4), we hypothesised that any associated phenotypes resulting from this suppression would be similarly detectable from day 7 onwards.

**Fig. 5:**
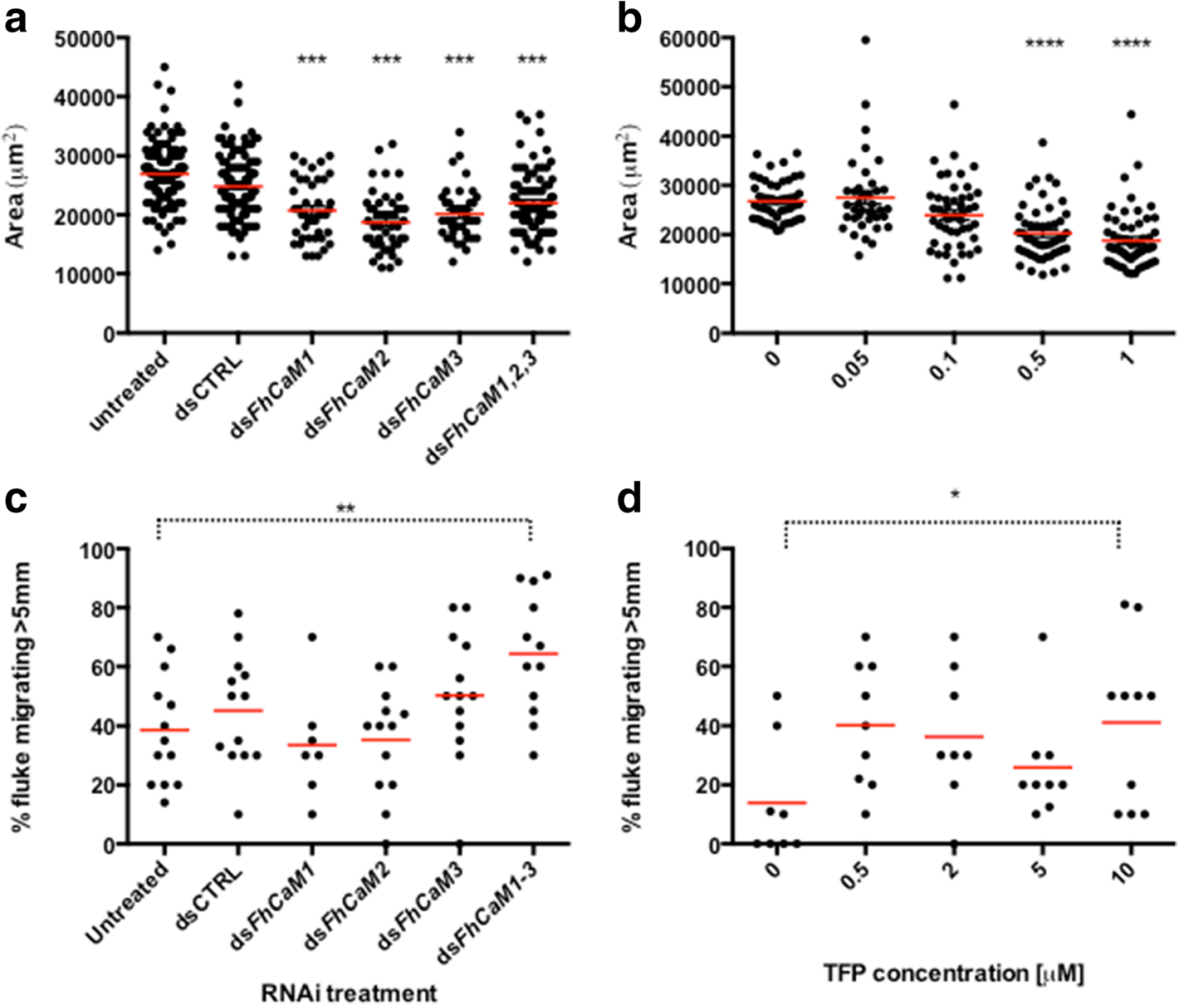
Calmodulin (CaM) RNA interference (RNAi) triggers aberrant growth and motility phenotypes in juvenile *Fasciola hepatica*, which are recapitulated by a CaM antagonising drug. **a** Fluke assayed for serum-stimulated growth (in RPMI + 20 % foetal bovine serum, FBS) at 7 days post double stranded (ds)RNA exposure show inhibited growth in individual and combinatorial *Fh*CaM dsRNA treatments *vs* controls; (**b**) this phenotype is recapitulated, over the same time period, by incubation in 0.5 μM or 1 μM trifluoperazine (TFP), an antagonist of CaM; (**c**) combinatorially-treated ds*Fh*CaM1-3 fluke show increased migration through an agar-based dispersal assay vs controls at 10 days post dsRNA exposure; (**d**) increased migration following CaM inhibition is also seen where worms are treated with TFP. In **a** and **b**, data points represent area measurements from individual worms. In **c** and **d**, each data point represents an individual assay plate, where 10 worms were measured per plate. Horizontal lines represent dataset means. *, *P* < 0.05; **, *P* < 0.01; ***, *P* < 0.001; ****, *P* < 0.0001

In order to measure impacts of CaM silencing on liver fluke growth, we employed an assay based on the growth of juvenile fluke, which can be triggered by the presence of bovine serum [36]. By digitally measuring the size (area occupied) of individual worms maintained in FBS-supplemented RPMI, we found that silencing *Fh*CaMs, either alone or in combination, inhibited serum-stimulated growth relative to controls (Fig. 5). Worms that were maintained for 7 days post dsRNA in vitro, demonstrated that *Fh*CaM RNAi juveniles grow more slowly than control treated juveniles (mean ± SEM worm area in each treatment: untreated, 26,952 ± 452 μm^2^, *n* = 124; dsCTRL, 24,820 ± 466 μm^2^, *n* = 122; ds*FhCaM1*, 20,714 ± 784 μm^2^, *n* = 42; ds*FhCaM2*, 18,714 ± 692 μm^2^, *n =* 49; ds*FhCaM3*, 20,150 ± 710 μm^2^, *n* = 40; ds*FhCaM1-3*, 21,966 ± 563 μm^2^, *n* = 88; Fig. 5). All four CaM RNAi treatments were statistically significant vs dsCTRL (*P* < 0.001). The supplementation of maintenance media with bovine serum was critical in revealing the growth phenotype associated with CaM disruption and emphasises the importance of optimising maintenance conditions for future *F. hepatica* experimentation. This is the first report of a growth phenotype revealed by RNAi in any flatworm parasite.

The rationale for investigating the impact of *Fh*CaM RNAi on fluke motility stemmed partly from our localisation data (see Fig. 2), where we suspected that some of the stained cells in the parenchyma would include muscle myocytons, combined with the well-documented role of CaM in regulating smooth muscle contraction [42]. If that was the case, we hypothesised that *Fh*CaM RNAi should affect the ability of worms to move and/or migrate (although CaM suppression may also have been expected to affect motility via disruption of Ca^2+^-induced synaptic vesicle release, or through more general induction of cellular stress associated with widespread Ca^2+^ disruption). Certainly, data from other systems link disruption of CaM function with hindered movement/motility, particularly in ciliated or flagellated organisms/cells [43]. Impacts of *Fh*CaM RNAi on fluke motility were quantified based on the ability of worms to migrate through an agar substrate, across a 5 mm radius from the point of origin. Performed at 10 days post dsRNA, this assay illustrated that combinatorially-treated ds*FhCaM1-3* (*P* < 0.01), but not individual ds*FhCaM* treatments, migrated significantly faster than untreated controls through this assay (proportion of worms escaping 5 mm radius: untreated, 38.6 ± 5.2 %, *n* = 13; dsCTRL, 45.2 ± 5.3 %, *n* = 13; ds*FhCaM1*, 33.6 ± 7.1 %, *n =* 7; ds*FhCaM2*, 35.3 ± 5.1 %, *n* = 13; ds*FhCaM3*, 50.2 ± 6.1 %, *n* = 13; ds*FhCaM1-3*, 64.3 ± 5.9 %, *n* = 12, *P* < 0.01; Fig. 5), i.e. worms exposed to a cocktail of three *Fh*CaM dsRNAs (ds*FhCaM1-3*) were hyperactive and migrated more quickly over a 3 h period than untreated controls. None of the individual ds*Fh*CaM treatments induced motor phenotypes that were significantly different from controls, suggesting that this phenotype may be the result of removing functional redundancy that may exist between the three fluke CaM genes.

### FhCaM RNAi phenotypes are recapitulated by the CaM antagonist, trifluoperazine

In order to further validate our RNAi-induced growth and motility phenotypes, we incubated juvenile worms in the presence of TFP, a compound with CaM antagonising activity. This compound (and another calmodulin antagonist, W7) has been shown to bind to *Fh*CaM1 and *Fh*CaM3 [20]. Additionally, CaM antagonists have been shown to partially block transformation in *S. mansoni* miracidia [13] and inhibit hatching of schistosome eggs [15]. Growth inhibition associated with *F. hepatica* CaM-RNAi was also seen in worms maintained in the presence of the CaM antagonist, TFP, consistent with the effects of this drug as reported in schistosome sporocysts [13]. Juvenile fluke were assayed after 7 days in the presence of TFP, with those treated with 0.5 and 1 μM showing significant reductions in size (*P* < 0.0001) compared to vehicle controls (untreated (no drug), 28,702 ± 890 μm^2^, *n* = 38; vehicle (DMSO) control, 26,775 ± 580 μm^2^, *n =* 50; 0.5 μM TFP, 20,276 ± 801 μm^2^, *n* = 50; 1 μM TFP, 18,776 ± 737 μm^2^, *n* = 60; Fig. 5).

**Fig. 6:**
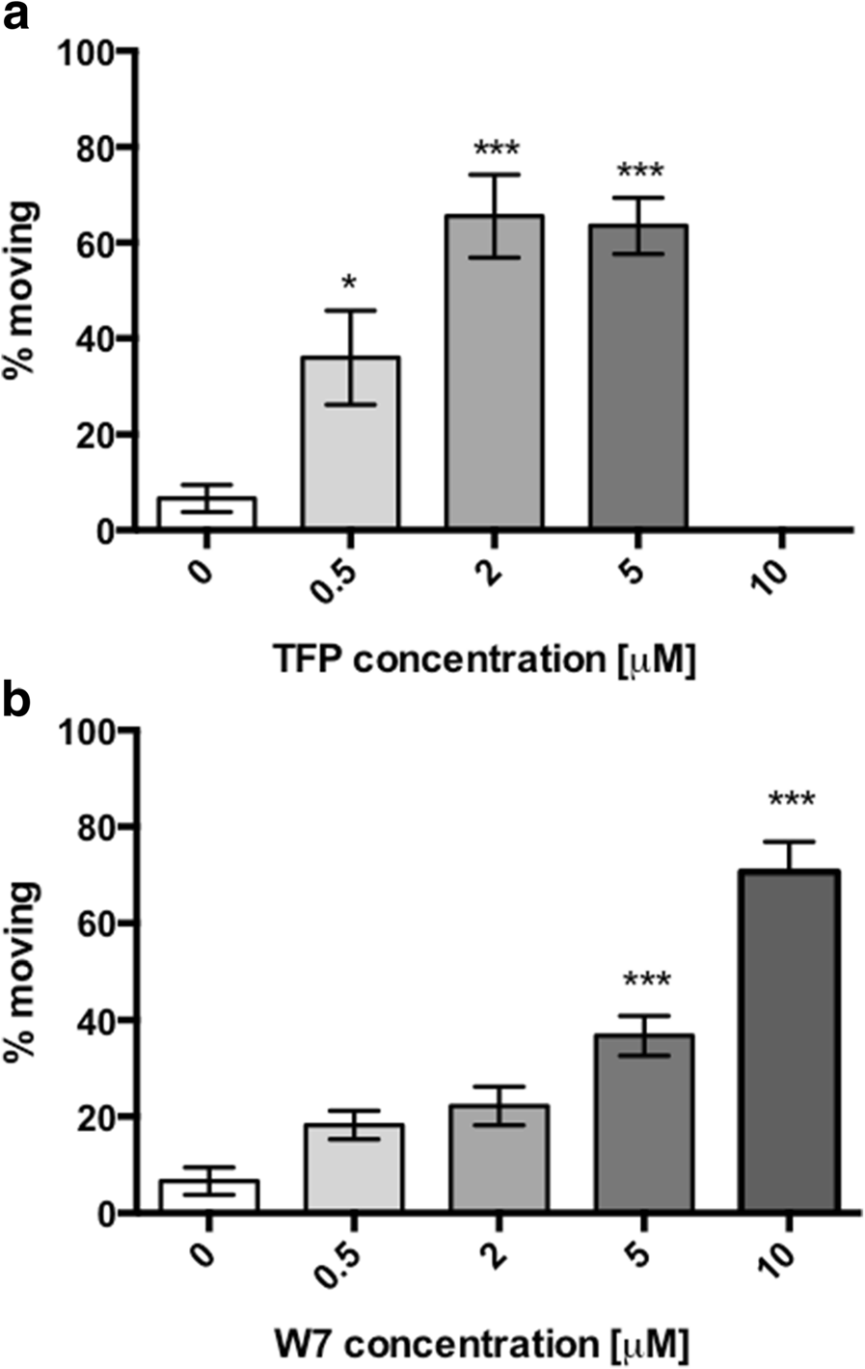
CaM antagonists stimulate motility in juvenile *Fasciola hepatica*. Increased motility (numbers moving) following CaM inhibition is seen when worms are treated with TFP (**a**) and W7 (**b**) over a period of 18 h. Each bar represents data from at least three RNAi treatment replicates, 20 worms per replicate. Statistical analyses were performed using One Way ANOVA with Dunnett’s post hoc test. *, *P* < 0.05; **, *P* < 0.01; ***, *P* < 0.001

TFP also increased the migratory capacity of juvenile fluke, in a manner similar to that displayed by *FhCaM1-3* RNAi juveniles. Following an 18 h exposure to concentrations of up to 5 μM TFP, we observed an increased proportion of worms displaying full motility during a 30 s observation in drug, compared to controls (visual analysis: vehicle (DMSO) control: 6.6 ± 2.8 % moving, *n* = 50; 5 μM TFP: 65.5 ± 5.9 % moving, *n* = 30, *P* < 0.001; Fig. 6). This effect was also evident in our agar assay, although only at 10 μM was the effect statistically significant vs controls (vehicle (DMSO) control, 13.9 ± 7.0 %, *n* = 8; 10 μM TFP, 41.1 ± 8.7 %, *n =* 10, *P* < 0.05; Fig. 5). W-7 hydrochloride also increased the proportion of worms moving under visual analysis (vehicle (DMSO) control: 6.6 ± 2.8 % moving, *n* = 50; 5 μM W-7: 36.8 ± 4.1 % moving, *n* = 30, *P* < 0.001; Fig. 6), but this difference did not translate into a statistically significant impact on fluke migration in the agar assay (data not shown).

As with the inhibited growth phenotype discussed above, this accelerated motility phenotype was recapitulated in the presence of the CaM-inhibiting drug, TFP. CaM RNAi has not, to our knowledge, been linked to increased motility in any other system to date, although TFP has been reported to increase spontaneous muscle contractions in the filarial nematode *Acanthocheilonema vitae* [44]*.* This increased motility phenotype might occur due to the disruption of intracellular Ca^2+^ dynamics in neuromuscular cells, either in muscle cells, by triggering aberrant contractility, or in neurones, by impacting on Ca^2+^-evoked synaptic release. We do not yet know how increased motility would impact on worm infectivity under in vivo conditions, although these experiments are currently in progress. While increased motility may not make an obvious therapeutic strategy, it is possible that the increased motility triggered by CaM RNAi might impact worm survival through increased energy expenditure. Alternatively, stunted growth and development as observed when *Fh*CaM function was disrupted by both molecular genetic and pharmacological means, does represent a desirable therapeutic outcome. This might reflect either: (i) *Fh*CaM’s direct involvement in controlling Ca^2+^ dynamics in dividing cells (Ca^2+^ fluxes have a well-established role in the cell cycle; see [45, 46]) where disrupting CaM function could directly inhibit cell division; or (ii) *Fh*CaMs are important housekeeping proteins required for normal cellular function [47], such that *Fh*CaM’s impact on growth occurs via a more general impact on cellular health/function in *Fh*CaM-expressing cells. Although the data presented in this study cannot further delineate between these possibilities, growth suppression could represent an appealing therapeutic outcome following *Fh*CaM-selective drug intervention.

## Conclusions

This work represents only the second published study to use gene-silencing methods to identify aberrant phenotypes that inform gene function in liver fluke parasites. We have demonstrated that CaMs are expressed widely in the parenchyma of the invasive juvenile life stage of *F. hepatica*. The development and application of novel *F. hepatica* assays demonstrated that CaMs are important for the normal growth and motility of NEJs. Additionally, the growth phenotype observed was only evident upon the addition of serum-stimulated development and we recommend the addition of serum to any further RNAi experiments in *F. hepatica.* It is worth noting that juvenile fluke growth in vitro is slower than that seen in vivo such that the growth phenotype associated with *Fh*CaM dysregulation could be markedly enhanced in vivo. While the increased motility effect does not implicate *FhCaM* as an obvious therapeutic target, the growth defects observed encourage further investigation. Future work will focus on deciphering functional differences in the roles of *Fh*CaM1, *Fh*CaM2 and *Fh*CaM3, particularly during development in vivo. The role of *Fh*CaMs in growth supports their consideration as control targets in liver fluke parasites.

## Additional file

Additional file 1:
**Figure S1.** (PDF 48 kb)
