# Supplementary material for: Calmodulin disruption impacts growth and motility in juvenile liver fluke
Source: Parasit Vectors. 2016 Jan 27;9:46. doi: 10.1186/s13071-016-1324-9 (PMC4730779; doi:10.1186/s13071-016-1324-9)
Supplement: Additional file 1: — Figure S1. (PDF 48 kb) [file 13071_2016_1324_MOESM1_ESM.pdf]

Supplementary material

Calmodulin disruption impacts growth and motility in juvenile liver fluke

Erin M. McCammick [a], Paul McVeigh [a], Paul McCusker [a], David J. Timson [a], Russell M. Morphew [b], Peter M. Brophy [b], Nikki J. Marks [a], Angela Mousley [a], Aaron G. Maule [a,*]

*Address correspondence to A G Maule, a.maule@qub.ac.uk

```
FhCaM1_scaff210          -ADQLTEEQIAEFKEAFSLFDKDGDGTITTKELGTVMRSLGQNPTEAELQDMINEVDADG
FhCaM1_scaff932_1        MADQLTEEQIAEFKEAFFLFDKDGDGTITTKELGTVMRSLGQNPTEAELQDMINEVDAD-
FhCaM1_scaff932_2        ------------------LFDKDGDGTITTKELGTVMRSLGQNPTEAELQDMINEVDAD-
FhCaM2_scaff2277         -VHHLSQDDIEVLRRAFSMYDQNGDGEIDATELKGVMWRLGCKPSDAEVREMIRKVDFD-
FhCaM3_scaff1560         MA---DFDMDHQLKEAFRNIDKNGDGFLSQDEISECLEMFGFKKNQSEYFMTLYDT---N
                                             *::*** :   *:   :  :* : .::*    : ..
                                  <      Linker Helix      >
FhCaM1_scaff932_1        GNGTIDFPEFLTMMARKMKDTDS-EEEIREAFRVFDKDGNGFISAAEL-RHVMTNLGEKL
FhCaM1_scaff932_2        GNGTIDFPEFLTMMARKMKDTDS-EEEIREAFRVFDKDGNGFISAAEL-RHVMTNLGEKL
FhCaM1_scaff210          GNGTIDFPEFLTMMARKMKDTDS-EEEIREAFRVFDKDGNGFISAAELRRHVMTNLGEKL
FhCaM2_scaff2277         DSGTINFPEFISMMVQKKRHAET-DANLRIAFQFFDRNGDGYISPEELRSVL-HKYRGNL
FhCaM3_scaff1560         NDGKISLDEFIKTTKRRVSDRDTTCALLRNTFRTIDQDHSGKITASELHKFFN-SSMGIV
                          .*.*.: **:.   ::  . ::    :* :*: :*:: .* *:  **   .  .    :

FhCaM1_scaff932_1        TDEEVDEMIREADIDGDGQVNYEEFVKMMTAK-
FhCaM1_scaff932_2        TDEEVDEMIREADIDGDGQVNYEEFVKMMTAK-
FhCaM1_scaff210          TDEEVDEMIREADIDGDGQVNYE----------
FhCaM2_scaff2277         DNNETEAIIKTVDTDRDGKLNYEEFLTLLRNQ-
FhCaM3_scaff1560         VPTTVEQWIEDHDKDGDNALSFEEFLEFAYEYL
                             .:  *.  * * *  :.:*
```

Supplementary Figure 1

Multiple amino acid sequence alignment of the *F. hepatica* calmodulin proteins encoded in the genome. An asterisk (*) indicates fully conserved/identical amino acids, a colon (:) indicates conservation between strongly similar amino acids, and a period (.) indicates conservation between weakly similar amino acids. Gray shading indicates the EF-hand domains (Ca-binding sites) and the brackets enclose the alpha-helix linker domain. These proteins are coded for at the following (inclusive) locations: FhCaM1_Scaff932_1, 149aa, coded at positions 97899-98345; FhCaM1_Scaff932_2, 131aa, coded at positions 98778-99170; FhCaM1_Scaff210, 139aa, coded at positions 222587-222763, 222801-222938, 227165-227266; FhCaM2_Scaff 2277, 148aa, coded at positions 89419-89246, 89207-89067, 74404-74303, 73311-73285; FhCaM3_Scaff1560, 146aa, coded at positions 175743-175609, 169742-169644, 168552-168394, 167886-167842. Note FhCaM2 and FhCaM3 are coded on the reverse strand of their respective scaffolds and the positions presented represent the complement strand.
